# High-Frequency Visual Stimulation Primes Gamma Oscillations for Visually Evoked Phase Reset and Enhances Spatial Acuity

**DOI:** 10.1093/texcom/tgab016

**Published:** 2021-03-03

**Authors:** Crystal L Lantz, Elizabeth M Quinlan

**Affiliations:** Department of Biology, University of Maryland, College Park, MD 20742, USA; Department of Biology, University of Maryland, College Park, MD 20742, USA; Neuroscience and Cognitive Science Program, University of Maryland, College Park, MD 20742, USA; Brain and Behavior Institute, University of Maryland, College Park, MD 20742, USA

**Keywords:** mouse, oscillations, photic tetanus, plasticity, visual cortex

## Abstract

The temporal frequency of sensory stimulation is a decisive factor in the plasticity of perceptual detection thresholds. However, surprisingly little is known about how distinct temporal parameters of sensory input differentially recruit activity of neuronal circuits in sensory cortices. Here we demonstrate that brief repetitive visual stimulation induces long-term plasticity of visual responses revealed 24 h after stimulation and that the location and generalization of visual response plasticity is determined by the temporal frequency of the visual stimulation. Brief repetitive low-frequency stimulation (2 Hz) is sufficient to induce a visual response potentiation that is expressed exclusively in visual cortex layer 4 and in response to a familiar stimulus. In contrast, brief, repetitive high-frequency stimulation (HFS, 20 Hz) is sufficient to induce a visual response potentiation that is expressed in all cortical layers and transfers to novel stimuli. HFS induces a long-term suppression of the activity of fast-spiking interneurons and primes ongoing gamma oscillatory rhythms for phase reset by subsequent visual stimulation. This novel form of generalized visual response enhancement induced by HFS is paralleled by an increase in visual acuity, measured as improved performance in a visual detection task.

## Introduction

Enduring changes in synaptic strength induced by specific frequencies of afferent stimulation are a primary mechanism for information storage in neural circuits. A wealth of data from brain slices demonstrates that high-frequency stimulation rapidly induces long-term potentiation (LTP) of excitatory synapses, while low-frequency stimulation induces synaptic long-term depression (Bliss and Lømo 1973; Dudek and Bear 1992; Mayford et al. 1995; Kirkwood et al. 1996). Repetitive stimulation delivered *in vivo* in animal models mirrors this dependence of bidirectional synaptic plasticity on the stimulation temporal frequency, with synaptic depression and potentiation induced by low and high frequency stimulation respectively (Nabavi et al. 2014; Rodríguez-Durán et al. 2017; O’Riordan et al. 2018). Furthermore, high-frequency direct stimulation of the visual thalamus or high-frequency photic tetanus induces long-lasting enhancement of visual responses (Zhang et al. 2000; Heynen and Bear 2001; Teyler et al. 2005; Clapp et al. 2006; Ross et al. 2008; Clapp et al. 2012; Eckert et al. 2013). Experiments in human subjects utilizing high-frequency sensory stimulation also demonstrate rapid enhancement of response amplitudes and lowering of perceptual thresholds in visual, auditory, and somatosensory systems (Ross et al. 2008; Kirk et al. 2010; Beste et al. 2011; Pegado et al. 2016; Marzoll et al. 2018; Brickwedde et al. 2020; Sumner et al. 2020). Enhancement of sensory responses induced by noninvasive sensory stimulation requires NMDA receptor activation, sharing the induction requirements for LTP (Dinse et al. 2003; Clapp et al. 2006). Frequency-dependent changes in neuronal activity and perception are also induced by transcranial stimulation.

Long-lasting enhancement of visual responses that require NMDA receptor activity is also induced in mouse primary visual cortex (V1) following daily repetition of low-frequency visual stimulation (LFS; Frenkel et al. 2006). This experience-dependent response plasticity includes an increase in the amplitude of the visually evoked potential (VEP) recorded in layer 4 of V1 and an increase in the peak firing rate of regular-spiking (RS) neurons (Cooke et al. 2015). Response potentiation induced by daily LFS is highly selective for the orientation, contrast, and spatial frequency of the visual stimulus used for induction. Indeed, response potentiation is not observed following rotation of the orientation of the visual stimulus by as little as 5 degrees (Cooke and Bear 2010). The time course and selectivity of visual response potentiation following daily LFS share many similarities with perceptual learning and LTP, including a requirement for sleep consolidation (Poggio et al. 1992; Zaehle et al. 2007; Aton et al. 2014). Interestingly, the selectivity of daily LFS visual response potentiation is lost following optogenetic suppression of parvalbumin expressing fast-spiking interneurons (FS INs; Kaplan et al. 2016). A reduction in putative FS IN excitability following deletion of the AMPAR-binding protein neuronal pentraxin 2 (NPTX2; aka NARP), which is highly enriched at excitatory synapses onto FS INs, inhibits the induction of visual response potentiation by a single bout of LFS (O’Brien et al. 1999; Xu et al. 2003; Chang et al. 2010; Gu et al. 2013). However, high temporal frequency visual stimulation (HFS, 20 Hz) can rescue visual response potentiation in NPTX2^−/−^ mice (Gu et al. 2013).

Sensory response amplitudes and perceptual detection thresholds reflect the interaction between stimulus-evoked neuronal activity and fluctuations in the cortical local field potential (LFP; Sauseng et al. 2007; Xing et al. 2012). In V1, the power of several bands of oscillatory activity in layer 4 increases in response to a familiar stimulus, suggesting that changes in oscillatory power encode visual stimulus familiarity (Kissinger et al. 2018). In concert, the phase of cortical oscillations regulates response magnitude and perception of incoming visual stimuli (Kim et al. 2007). The temporal frequency of incoming auditory and somatosensory stimulation can also impact sensory perception through entrainment of ongoing cortical oscillations (ten Oever et al. 2017; Brickwedde et al. 2020). Indeed, increased power and synchronization of high-frequency cortical oscillations are thought to underlie improvements in stimulus detection and memory encoding by attention and motivation (Montgomery and Buzsáki 2007; Jutras et al. 2009; Lisman 2010). Thus, changes in the time-locked evoked response, as well as the magnitude and phase of ongoing cortical oscillations, are candidate mechanisms for synaptic plasticity evoked by repetitive sensory stimulation (Park et al. 2016; Howe et al. 2017; Brickwedde et al. 2019).

Although the temporal parameters of visual stimulation play a decisive role in the induction of plasticity of visual responses and perceptual thresholds, it is not known how the temporal frequency of visual stimulation differentially recruits activity in V1 neuronal circuitry. Here, we directly compare the impact of two distinct temporal frequencies of repetitive visual stimulation on long-lasting changes in visual response magnitude and visual acuity in the mouse. We find that a single bout of LFS is sufficient to induce a visual response potentiation that is restricted to visual cortex layer 4 and the familiar visual stimulus. In contrast, a single bout of HFS is sufficient to induce visual response potentiation throughout V1 that transfers to novel stimuli. HFS induces a long-lasting suppression of the output of FS INs and sensitizes cortical gamma oscillations to phase reset by all subsequent visual stimuli. This general enhancement of visual response strength following HFS is paralleled by improved visual acuity revealed by performance in a visual detection task.

## Materials and Methods

### Animals

Experiments utilized equal numbers of male and female adult (postnatal day 60–90) C57BL/6J mice (Jackson Lab, Bar Harbor, ME). Subjects were housed on a 12:12 h dark:light cycle with food and water ad libitum. Experiments were initiated ~6 h into the light phase. All procedures conformed to the guidelines of the University of Maryland Institutional Animal Care and Use Committee and in accordance with the guidelines published in the NIH Guide for the Care and Use of Laboratory Animals. Sample sizes were determined by power analysis of previous studies quantifying the effect of visual experience on visual response amplitudes.

### Electrophysiology

House-made 1.2-mm length 16-channel shank electrodes were implanted into binocular V1 (from Bregma: posterior, 2.8 mm; lateral, 3.0 mm; ventral, 1.2 mm), under anesthesia with 2.5% isoflurane in 100% O_2_, as described (Murase et al. 2017; Bridi et al. 2018). Multichannel electrode arrays were built as previously described (Zold and Hussain Shuler 2015; Murase et al. 2017). Briefly, platinum iridium wires with a diameter of 15 μm (California Fine Wire) were glued into a flat array and cut at an angle to obtain a spacing of approximately 75 μm. Each electrode tip was electroplated with gold (SIFCO Applied Surface Concepts, Supplementary Fig. S1A). Subjects received a single dose of carprofen (5 mg/kg, SQ) for post-surgical analgesia after the return of the righting reflex. One week after surgery and 1 day before electrophysiological recordings, subjects were habituated for 45 min to head restraint. Broadband electrophysiological data were collected from awake head-fixed mice, using RZ5 bioamp processor and RA16PA preamp (Tucker Davis Technologies, TDT). Multiunit waveforms were sorted into single units (SUs) using an automatic Bayesian clustering algorithm in OpenSorter (TDT) as described (Murase et al. 2017). SUs were processed in MATLAB and classified as RS neurons (presumptive excitatory) or putative FS INs (presumptive inhibitory) based on waveform slope 0.5 ms after the trough, time between trough and peak, and the ratio of trough to peak height (Niell and Stryker 2008, Supplementary Fig. S1E,F). VEPs and SUs were assigned to cortical layer based on LFP waveform shape and current source density calculated with single site spacing from the laminar array (Mitzdorf 1985; Guo et al. 2017, Supplementary Fig. S1B,C).

### Visual Stimulation

Visual stimuli were presented to both eyes simultaneously using MATLAB (Mathworks) with Psychtoolbox extensions (Brainard 1997; Pelli 1997). Prior to visual stimulation each day, mice passively viewed a gray 26 cd/m^2^ screen for 200 s to acquire spontaneous activity. Visually evoked responses were recorded in response to 200 s of 0.05 cycles per degree, 100% contrast, square-wave gratings reversing at 2 Hz (LFS). Responses were averaged over 1 second. Subjects received 2 Hz LFS stimulation for the acquisition of baseline, initial VEPs. A subset of subjects then received visual stimulation at 20 Hz (HFS). VEPs were acquired on day 2 in response to 2 Hz stimulation in response to familiar and novel stimulus orientations and compared back to the baseline response to 2 Hz stimulation.

### Data Analysis

Spike rates of sorted SUs were calculated as the average over each 1-s epoch (200 s of continuous visual stimulation). Peristimulus-time histograms (PSTHs) were calculated for each SU using 5-ms bins and smoothed with a Gaussian kernel (Kissinger et al. 2018). To examine changes in oscillatory power by frequency, PSTHs were *z*-scored and filtered from 1 to 100 Hz using a sliding frequency window via a bandpass elliptic filter with a span of 3 Hz in MATLAB. The analytic signal of band-passed PSTHs was calculated using a Hilbert transform, and the absolute value was used to calculate power within each frequency band.

VEPs were calculated as the trough to peak amplitude of the average of 1-s LFP epochs during visual stimulation in MATLAB, as described (Murase et al. 2017). To examine changes in oscillatory power by frequency and the reliability of incoming visual stimulation to reset the phase of ongoing oscillations (intertrial phase consistency, ITPC, a time-locked measure of oscillatory phase), spontaneous and evoked LFPs were *z*-scored and then convolved with complex Morlet wavelets from 1 to 100 Hz using a 3 Hz window (Fiebelkorn et al. 2018). The wavelet cycle width varied with filtered frequency (1–10 Hz = 2 cycles, 11–14 Hz = 3 cycles, 15–20 Hz = 4 cycles, 21–100 Hz = 5 cycles). The absolute value of the complex vector was used to calculate oscillatory power. Power was averaged across trials for each subject, and activity is reported as percent change in power relative to spontaneous activity recorded on the first day prior to experimental manipulation (experimental – baseline/baseline × 100). Averaged percent change in evoked power was calculated during the 100–200 ms post stimulus onset, and binned oscillatory activity was averaged from this window. Oscillatory bins were defined as follows: delta 1–4 Hz, theta 4–8 Hz, alpha 8–13 Hz, beta 13–30 Hz, and gamma 30–100 Hz. The second half of the complex vector was normalized, averaged, and the absolute value was used to calculate ITPC. ITPC was binned by oscillatory frequency. Utilizing the calculated oscillatory phase of the LFP, the phase of each frequency for each SU was calculated and then averaged as spike-phase consistency.

### Behavior

Psychophysical measurements of spatial acuity were obtained through with performance in a two-alternative forced choice visual detection task. Task training and testing utilized a Bussey–Saksida Touch Screen Chamber (Lafayette Instruments; Horner et al. 2013) with custom plexiglass inserts. An opaque insert divided the LCD touch screen into two vertical halves, for simultaneous display of the correct and incorrect visual stimuli. A transparent plexiglass insert, parallel to and 6 cm from the touch screen, with two 5 × 7 cm swing-through doors, defined the choice point for the calculation of visual stimulus spatial frequency (Fig. 7*A*). Naïve adult mice were trained to associate a high contrast (100%), low spatial frequency (0.05 cycles/degree), 45° sinusoidal grating (positive stimulus) with positive reinforcement (strawberry milk and tone, 3 kHz, 0.5 s), and a gray image of equal luminance (32 cd/m^2^; negative stimulus) with negative reinforcement (tone, 400 Hz, 2 s). Subjects were food deprived for 20 h a day, with 4 h of ad libitum food access at the end of each training session. A training session consisted of 30 trials, or 45 min. To begin a trial, the subject nose-poked in the illuminated liquid reward tray at the rear of the chamber, to trigger the presentation of positive and negative visual stimuli. Contact with the touch screen turned the visual stimulus off. Choosing the positive stimulus resulted in a liquid reward, and choosing the negative stimulus resulted in negative reinforcement and a 30-s timeout period. Trials were separated by a 10-s intertrial interval, followed by illumination of the liquid reward tray to signal the beginning of a new trial. Criterion was defined as 25/30 correct trials within 45 min (83% correct). After reaching criterion, acuity testing was initiated. In acuity testing, the positive stimulus was rotated to a novel orientation (45° + 15°). Following successful completion (≥70%) of a block of 10 trials, the spatial frequency was increased incrementally (0.05 cpd steps). The highest spatial frequency with performance of ≥70% correct choices is defined as spatial acuity. Following assessment of initial acuity, subjects were randomly divided into two groups, 50% viewed 200 s of LFS and 50% viewed 200 s of HFS, at a novel orientation, and were returned to their home cage. Twenty-four hours after visual stimulation, acuity was tested at a familiar stimulus orientation (used for LFS or HFS) and at a novel orientation, with test order randomized. Subjects were then returned to food and water ad libitum in the mouse colony.

### Statistics

Statistical analysis was completed using JASP (JASP Stats). Repeated measures analysis of variance (RANOVA) was used to compare LFP data from three time points within the same subject, including VEP, oscillatory power, and ITPC, followed by a Bonferroni post hoc when appropriate. For SU recordings, we did not assume that we were recording from the same unit over multiple days; therefore, unpaired Student’s *t*-test was used to compare two groups and a one-way ANOVA was used to compare three groups, followed by a Tukey post hoc when appropriate. A multivariate ANOVA (MANOVA) with a Bonferroni post hoc, when appropriate, was used to compare oscillatory data consisting of two time points and multiple frequency bands. To compare the change in oscillatory power within subjects, we employed a one-sample Student’s *t*-test with a Bonferroni correction for multiple comparisons. In text, *n* is reported as the total number of subjects, followed by the total number of SUs. Exact *P* values are reported in the text, except when *P* < 0.001.

## Results

### Plasticity of Visual Responses Is Dependent on Visual Stimulation Frequency

**Figure 1:**
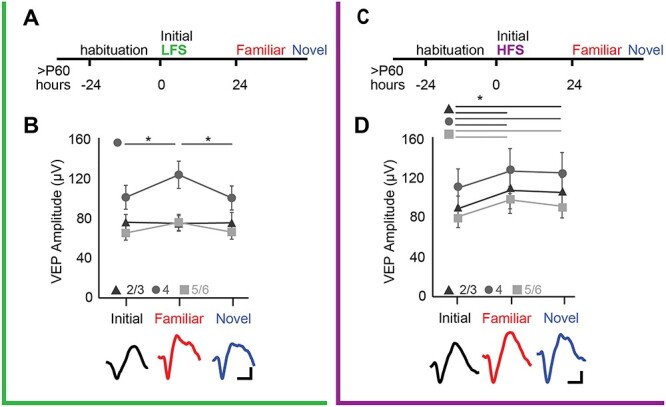
LFS and HFS differentially impact the location and generalization of visual response potentiation. (*A*) Experimental timeline: naïve adult subjects (>P60) received low-frequency visual stimulation (LFS, green: 200 presentations of 0.05 cpd, 100% contrast gratings, 60° orientation, 2 Hz). The initial response is reported as baseline VEP. After 24 h, VEPs were evoked by visual stimuli with familiar and novel orientations presented at 2 Hz. (*B*) Significant increase in VEP amplitude in layer 4 (circles) in response to familiar, but not novel, stimuli (RANOVA _(df, 2, 15)_, *F* = 9.13, *P* < 0.001; ^*^Bonferroni post hoc *P* < 0.05; *n* = 16 subjects). Bottom: Representative example of layer 4 VEP in response to initial (black), familiar (red), and novel (blue) stimuli. Cal = 20 μV, 50 ms. (*C*) Timeline, as in *A*, except that naïve adult subjects received high-frequency visual stimulation (HFS, purple: 200 presentations of 0.05 cycle per degree, 100% contrast grating, 30° orientation, 20 Hz) after initial assessment of baseline VEP. After 24 h, VEPs were evoked by visual stimuli with familiar and novel orientations presented at 2 Hz. (*D*) Significant increase in VEP amplitudes in layers 2/3 (triangles), 4 (circles), and 5/6 (squares) in response to familiar and novel stimuli (RANOVA _(df, 2, 10)_, layer 2/3: *F* = 5.25, *P* = 0.015, layer 4: *F* = 7.31, *P* = 0.004; layer 5/6: *F* = 11.41, *P* < 0.001; ^*^Bonferroni post hoc *P* < 0.05; *n* = 11 subjects). Bottom: Representative example of layer 4 VEP in response to initial (black), familiar (red), and novel (blue) stimuli. Cal = 20 μV, 50 ms.

To examine the impact of the temporal frequency of repetitive visual stimulation on visual response plasticity, we examined the magnitude of VEPs simultaneously in all layers of the primary visual cortex (V1). We focused on the long-term effects of one bout of visual stimulation, as previously described (Gu et al. 2013; Montey et al. 2013). Head-fixed awake mice viewed 200 s of square-wave gratings (0.05 cycles per degrees, 100% contrast, 60° orientation) delivered at low frequency (LFS, 2 Hz) or high frequency (HFS, 20 Hz, Fig. 1*A*,*C*), and VEPs were averaged over 1 s. Twenty-four hours after LFS or HFS, VEPs were recorded a second time in response to visual stimulation with a familiar (60°) and novel (150°) orientation. Long-lasting visual response potentiation was induced by both protocols, and the location and generalization of visual response potentiation were determined by the stimulus temporal frequency. Twenty-four hours after a single bout of LFS, the amplitude of the layer 4 VEP was significantly increased in response to familiar (60°), but not novel (150°) visual stimulus orientations, mimicking the stimulus selectivity of response potentiation induced by LFS over multiple days (Frenkel et al. 2006; *n* = 16, RANOVA_(df, 2, 15)_, Bonferroni post hoc, *F* = 9.13, *P* < 0.001; initial vs. familiar: *P* = 0.023, Fig. 1*B*).

In contrast, 24 h after a single bout of HFS, VEP amplitudes were significantly increased in all layers of V1 in response to both familiar (60°) and novel (150°) visual stimulus orientations (*n* = 11, RANOVA _(df, 2, 10)_, Bonferroni post hoc, layer 2/3: *F* = 5.25, *P* = 0.015, initial vs. familiar: *P* = 0.023, initial vs. novel: *P* = 0.032; layer 4: *F* = 7.31, *P* = 0.004, initial vs. familiar: *P* = 0.038, initial vs. novel: *P* = 0.005; layer 5/6: *F* = 11.41, *P* < 0.001, initial vs. familiar: *P* = 0.005, initial vs. novel: *P* = 0.022, Fig. 1*D*). Twenty-four hours after HFS, amplitudes evoked by visual stimuli of novel spatial frequencies were also increased (Supplementary Fig. S2). To control for the delivery of 2 Hz stimulation used for assessment of baseline VEPs prior to delivery of 20 Hz stimulation, we also examined VEP amplitudes in subjects that received 20 Hz stimulation prior to 2 Hz stimulation. Generalized visual response potentiation was observed throughout V1 if HFS (20 Hz) preceded or followed the assessment of initial baseline VEP amplitudes (between-subjects RANOVA _(df 1,12)_ layer 2/3: *F* = 0.001, *P* = 0.97; layer 4: *F* = 0.010, *P* = 0.92; layer 5: *F* = 0.00003, *P* = 0.995, data not shown). Notably, we observe no evidence of immediate potentiation of VEPs in response to HFS, as VEP amplitudes acquired with subsequent baseline stimulation (2 Hz) were similar to unstimulated controls (Naive initial: layer 2/3:81.12 ± 11.47 μV; layer 4: 110.02 ± 19.34 μV; layer 5: 80.80 ± 10.92 μV; initial following HFS: layer 2/3: 76.66 ± 7.61 μV; layer 4: 115.49 ± 25.12 μV; layer 5: 79.52 ± 17.91 μV. RANOVA_(df, 1, 12)_, between subjects, *F* = 0.00012, *P* = 0.997). HFS and LFS were matched for stimulus duration and not number of stimulus reversals, as previous work demonstrates that repetitive LFS over consecutive day induces stimulus-specificity response potentiation in layer 4 (Frenkel et al. 2006; Cooke and Bear 2010). Thus, response potentiation induced by a single bout of LFS is localized and stimulus-specific, while HFS-induced response potentiation is global and transfers to novel stimuli.

### Visual Stimulus Frequency Acutely Modulates Oscillatory Power, Phase, and LFP–Spike Coupling in V1

**Figure 2:**
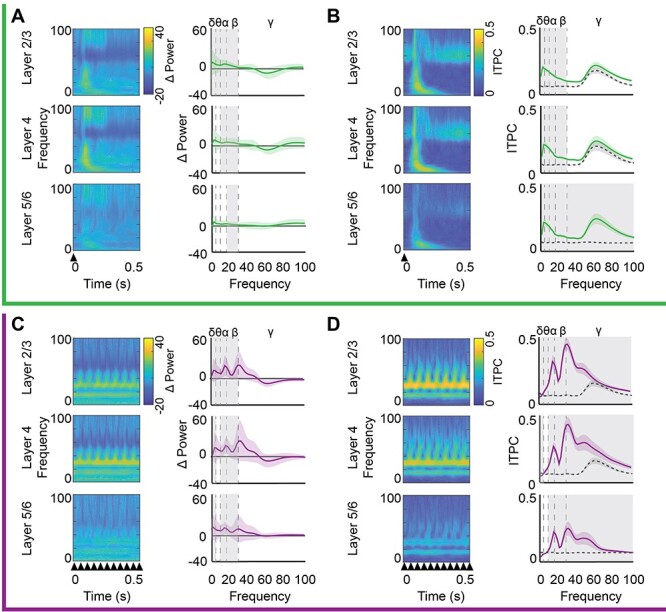
Distinct acute impact of LFS and HFS on oscillatory power and evoked phase reset. Top, green, during LFS: (*A*) Left: Average oscillatory power (heat map) from 0 to 100 Hz (3 Hz bins; *y*-axis) over time (*x*-axis) by cortical layer during LFS. Power was normalized to spontaneous activity in response to 26 cd/m^2^ gray screen. Right: Significant increase in average α and β power across all cortical layers, δ power in layers 2/3 and 4, and θ power in layers 2/3 and 5/6 during LFS (one-sample *t*-test, gray highlight = *P* < 0.05, *n* = 16). Power binned by frequency band (δ: 1–4, θ: 4–8, α: 8–13, β: 13–30, γ: 30–100 Hz). (*B*) Left: Average ITPC during LFS (ITPC; heat map) from 0 to 100 Hz (in 3 Hz bins; *y*-axis) over time (*x*-axis), trial averaged. Right: A significant increase in ITPC for all frequencies in layer 2/3 (MANOVA_(df, 1, 5)_, *F* = 15.94, *P* < 0.001), and all frequencies below gamma in layers 4 (MANOVA_(df, 1, 15)_, *F* = 15.98, *P* < 0.001) and 5/6 (MANOVA_(df, 1, 15)_, *F* = 11.10, *P* < 0.001) during LFS (solid line), relative to spontaneous activity (dashed line). Gray highlight = Bonferroni post hoc *P* < 0.05; *n* = 16 subjects. Bottom, purple, during HFS: (*C*) Left: Average oscillatory power (heat map) from 0 to 100 Hz (3 Hz bins; *y*-axis) over time (*x*-axis) by cortical layer during HFS, normalized as in *A*. Right: A significant increase in average δ, α, and β power in all cortical layers and in θ power in layer 2/3 during HFS (one-sample *t*-test, gray highlight = *P* < 0.05, *n* = 11). (*D*) Left: Average ITPC during HFS (ITPC; heat map) from 0 to 100 Hz (in 3 Hz bins; *y*-axis) over time (*x*-axis), trial averaged as in *B*. Right: Significant changes in ITPC in all cortical layers, including a significant increase in visually driven phase reset in α and β oscillations (layer 2/3: MANOVA_(df, 1, 10)_, *F* = 14.14, *P* < 0.001; layer 4: MANOVA_(df, 1, 10)_, *F* = 12.284, *P* < 0.001; layer 5: MANOVA_(df, 1, 10)_, *F* = 9.95, *P* = 0.0017), and a significant decrease in the δ oscillation in all layers, during HFS (purple) relative to spontaneous activity (dashed line). Gray highlight = Bonferroni post hoc *P* < 0.05; *n* = 11 subjects. Stimulus onset denoted by arrowheads.

The VEP amplitude reflects the interaction between stimulus-evoked synaptic potentials and ongoing fluctuations in the cortical LFP. Changes in VEP amplitude could therefore reflect changes in the power or phase of LFP oscillations. To ask how the power of LFP oscillations was impacted during LFS and HFS, we normalized the absolute value of the complex Morlet wavelet convolved LFP during visual stimulation to prestimulation spontaneous activity (equal luminance gray screen; 26 cd/m^2^). LFS and HFS induced similar changes in LFP oscillatory power. During LFS, low-frequency (alpha and beta bands) LFP oscillatory power increased significantly in all cortical layers (*n* = 16, one-sample *t*-test. Layer 2/3—α: *t* = 4.44, *P* < 0.001; β: *t* = 4.39, *P* < 0.001. Layer 4—α: *t* = 2.37, *P* = 0.015, β: *t* = 2.31, *P* = 0.024. Layer 5—α: *t* = 4.07, *P* < 0.001; β: *t* = 2.27, *P* = 0.019, Fig. 2*A*, Supplementary Fig. S3A,B). During HFS, low frequency (delta, alpha, and beta bands) power increased significantly in all layers (*n* = 11, one-sample *t*-test, layer 2/3—δ: *t* = 2.42, *P* = 0.019; α: *t* = 3.21, *P* = 0.005; β: *t* = 3.57, *P* = 0.003; layer 4—δ: *t* = 2.11, *P* = 0.031; α: *t* = 2.94, *P* = 0.008; β: *t* = 2.82, *P* = 0.009; layer 5—δ: *t* = 2.31, *P* = 0.023; α: *t* = 1.93, *P* = 0.042; β: *t* = 1.92, *P* = 0.043; Fig. 2*C*, Supplementary Fig. S3C,D).

**Figure 3:**
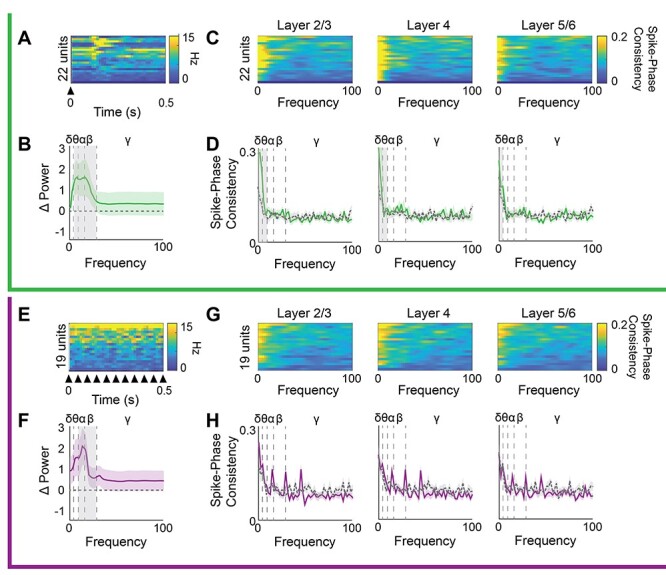
Distinct acute impact of LFS and HFS on FS IN oscillatory power and LFP phase synchrony. Top, green, during LFS: (*A*) Heat map depicting PSTH for each FS IN. (*B*) Average change in oscillatory power of FS INs during LFS, binned by frequency band (δ: 1–4, θ: 4–8, α: 8–13, β: 13–30, γ: 30–100 Hz, *n* = 16 subjects). A significant increase in the power of θ, α, and β oscillations in FS IN during LFS (one-sample *t*-test with Bonferroni correction, gray highlight = *P* < 0.05). (*C*) Heat map of FS IN spike–LFP phase consistency during LFS for each FS IN compared with LFP of each layer. (*D*) FS IN spike–LFP phase consistency during LFS (green) compared with spontaneous activity (gray), by frequency (average ± SEM). LFS increases FS IN spike–phase consistency with δ oscillations in all cortical layers and with θ in layers 2/3 and 4 (two-sample *t*-test, gray highlight = *P* < 0.05, *n* = 16 subjects). Bottom, purple, during HSF: (*E*) Heat map depicting PSTH for each FS IN. (*F*) Average change in oscillatory power of FS IN spiking during HFS, power binned by frequency band (δ 1–4, θ: 4–8, α: 8–13, β: 13–30, γ: 30–100 Hz, *n* = 11 subjects). A significant increase in the power of α and β oscillations in FS INs during HFS (one-sample *t*-test, with Bonferroni correction, gray highlight = *P* < 0.05). (*G*) Heat map of FS IN spike–LFP phase consistency during HFS for each FS IN compared with LFP of each layer. (*H*) Average ± SEM FS IN spike–LFP phase consistency during HFS (purple) compared with spontaneous (gray) by frequency. HFS does not change FS IN spike–phase consistency in any cortical layer (*n* = 11 subjects).

To ask how the temporal frequency of visual stimulation impacted the phase of ongoing LFP oscillations, we convolved the LFP signal with a complex Morlet wavelet and calculated the angle of the resultant complex output. ITPC, which ranges from 0 (if phase was random, and not reset by incoming visual input) to 1 (if phase was reset similarly in all trials), was calculated from the time-locked phase and compared with the ITPC during pre-stimulation spontaneous activity. LFS and HFS had differential effects on the phase reset of ongoing LFP oscillations. LFS increased phase reset for low-frequency oscillations (delta, theta, alpha, and beta) in all cortical layers and increased gamma phase reset in layer 2/3 (*n* = 16 subjects, layer 2/3: MANOVA _(df, 1, 5)_*F* = 15.94, *P* < 0.001, δ: *F* = 36.41, *P* < 0.001, θ: *F* = 75.21, *P* < 0.001, α: *F* = 88.20, *P* < 0.001, β: *F* = 42.22, *P* < 0.001, γ: *F* = 4.277, *P* = 0.047. Layer 4: MANOVA _(df, 1, 5)_*F* = 15.98, *P* < 0.001, δ: *F* = 45.01, *P* < 0.001, θ: *F* = 56.15, *P* < 0.001, α: *F* = 65.05, *P* < 0.001, β: *F* = 39.86, *P* < 0.001. Layer 5/6: MANOVA _(df, 1, 5)_*F* = 11.10, *P* < 0.001, δ: *F* = 20.59, *P* < 0.001, θ: *F* = 51.07, *P* < 0.001, α: *F* = 46.05, *P* < 0.001, β: *F* = 35.09, *P* < 0.001; Fig. 2*B*). In contrast, HFS significantly increased phase reset of intermediate frequencies (alpha and beta) in all cortical layers and increased gamma reset in layers 2/3 and 4. Interestingly, HFS decreased delta reset in all cortical layers, with no changes observed in the phase of theta oscillations (*n* = 11 subjects, layer 2/3: MANOVA _(df, 1, 5)_*F* = 10.46, *P* < 0.001, δ: *F* = 7.76, *P* = 0.011, α: *F* = 16.68, *P* < 0.001, β: *F* = 43.33, *P* < 0.001, γ: *F* = 10.63, *P* = 0.004. Layer 4: MANOVA _(df, 1, 5)_*F* = 11.26, *P* < 0.001, δ: *F* = 10.09, *P* = 0.005, α: *F* = 28.28, *P* < 0.001, β: *F* = 48.15, *P* < 0.001, γ: *F* = 5.60, *P* = 0.028. Layer 5/6: MANOVA _(df, 1, 5)_*F* = 6.46, *P* = 0.002, δ: *F* = 5.84, *P* = 0.025, α: *F* = 24.25, *P* < 0.001, β: *F* = 34.24, *P* < 0.001; Fig. 2*D*). The output of putative fast spiking interneurons (FS INs) can regulate LFP amplitudes by influencing the generation of theta rhythms and the power and synchrony of gamma rhythmicity (Cardin et al. 2009; Sohal et al. 2009; Stark et al. 2013). To quantify the pattern and strength of the spiking output of individual FS INs during LFS and HFS, we calculated the oscillatory power of the post-stimulus time histogram from SU activity. Oscillatory power during visual stimulation was normalized to pre-stimulation spontaneous activity. The temporal frequency of the visual stimulus was reflected in the output of FS INs, as LFS increased the power of low (4–8 Hz) as well as mid-frequency oscillations (7–30 Hz, *n* = 16 subjects, 23 units. One-sided *t*-test, θ: *t* = 2.22, *P* = 0.016, α: *t* = 2.04, *P* = 0.024, β: *t* = 1.84, *P* = 0.036; Fig. 3*A*,*B*). In contrast, HFS increased the power of higher frequency oscillations (13–30 Hz, *n* = 11 subjects, 19 units. One-sided *t*-test, α: *t* = 1.81, *P* = 0.039, β: *t* = 2.41, *P* = 0.011; Fig. 3*E*,*F*). To quantify the coherence of FS IN activity with ongoing LFP oscillations, we utilized the time-locked LFP phase to calculate the consistency of FS IN spiking within each oscillatory frequency band during LFS and HFS. LFS significantly increased FS IN firing phase consistency with delta oscillations in all cortical layers and theta oscillations in layers 2/3 and 4 (Student’s *t*-test, *n* = 16 subjects, 22 units, layer 2/3: δ: *t* = 3.63, *P* < 0.001, θ: *t* = 3.66, *P* < 0.001; layer 4: δ: *t* = 8.10, *P* < 0.001, θ: *t* = 3.87, *P* < 0.001; layer 5: δ: *t* = 2.02, *P* = 0.025, Fig. 3*C*,*D*). Unexpectedly, no significant differences in FS IN firing phase consistency were observed during HFS (Fig. 3*G*,*H*).

### Changes in Oscillatory Power Do Not Predict Visual Response Potentiation

To ask if LFS and HFS induce long-lasting changes in LFP oscillations, we examined the power of spontaneous oscillations and the response to the familiar (60° orientation) and novel (150° orientation) visual stimuli 24 h after repetitive visual stimulation. We utilized the absolute value of the Morlet wavelet convolved LFP in the time window of maximal visually evoked activity (100–200 ms after stimulus reversal). Twenty-four hours after HFS, delta and theta power of spontaneous activity were significantly decreased (Supplementary Figs S4D,E and S5B), with no change in spontaneous power 24 h after LFS (Supplementary Figs S4A,B and S5A). Thus, HFS induced long-lasting changes in ongoing cortical rhythms.

**Figure 4:**
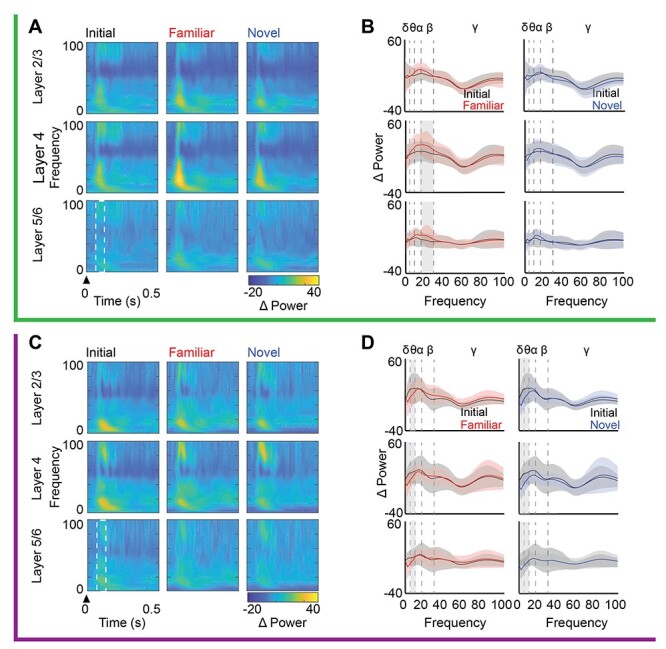
LFS and HFS differentially impact oscillatory power during subsequent visual stimulation. Top, green, after LFS: (*A*) Left: Average oscillatory power (heat map) from 0 to 100 Hz (3 Hz bins; *y*-axis) over time (*x*-axis) by cortical layer during initial LFS and in response to familiar and novel visual stimuli 24 h after LFS. Power is normalized to pre-experimental spontaneous activity acquired during viewing of a 26 cd/m^2^ gray screen. Arrowhead indicates stimulus onset and white box indicates time window for assessment of change in oscillatory power (100–200 ms after stimulus onset). (*B*) Change in oscillatory power (average ± SEM), binned by frequency band (δ: 1–4, θ: 4–8, α: 8–13, β: 13–30, γ: 30–100 Hz) during presentation of initial (black), familiar (red), and novel (blue) visual stimuli. In layers 4 and 5/6, a significant increase in average β power in response to familiar (red), but not novel (blue) visual stimuli relative to initial, in subjects that received LFS (black, RANOVA _(df, 2, 15)_, layer 4: *F* = 6.51, *P* = 0.004; layer 5/6: *F* = 3.92, *P* = 0.031). Gray highlight = Bonferroni post hoc *P* < 0.05; *n* = 16 subjects. Bottom, purple, after HFS: (*C*) Average oscillatory power (heat map) from 0 to 100 Hz (3 Hz bins; *y*-axis) over time (*x*-axis) by cortical layer during initial HFS and in response to presentation of familiar and novel visual stimulus orientations 24 h after HFS. Power was normalized as in *A*. Arrowhead indicates stimulus onset and white box indicates time window for assessment of change in oscillatory power (100–200 ms after stimulus reversal). (*D*) Change in oscillatory power (average ± SEM), binned by frequency band during presentation of initial (black), familiar (red), and novel (blue) visual stimuli. A decrease in average θ power in response to novel (blue) and familiar (red) visual stimuli, in all layers in subjects that received HFS (RANOVA _(df, 2, 10)_, layer 2/3: *F* = 8.63, *P* = 0.002; layer 4: *F* = 8.47, *P* = 0.002; layer 5: *F* = 7.936, *P* = 0.003). Gray highlight = Bonferroni post hoc *P* < 0.05; *n* = 11 subjects.

Visually evoked changes in LFP power were also different following LFS and HFS. Twenty-four hours after LFS, presentation of visual stimuli with familiar, but not novel orientations significantly increased beta power (13–30 Hz) in layers 4 and 5/6 (*n* = 16, RANOVA _(df, 2, 15)_, Bonferroni post hoc. Layer 4: *F* = 6.51, *P* = 0.004, initial vs. familiar: *P* = 0.004. Layer 5/6: *F* = 3.92, *P* = 0.031, initial vs. familiar: *P* = 0.027; Fig. 4*A*,*B*). In contrast, 24 h after HFS, presentation of visual stimuli with familiar or novel orientations significantly decreased theta power in all cortical layers (*n* = 11, RANOVA _(df, 2, 10)_ with Bonferroni post hoc. Layer 2/3: *F* = 8.633, *P* = 0.002; initial vs. familiar: *P* = 0.015, initial vs. novel: *P* = 0.003. Layer 4: *F* = 8.47, *P* = 0.002; initial vs. familiar: *P* = 0.016, initial vs. novel: *P* = 0.003. Layer 5/6: *F* = 7.936, *P* = 0.003; initial vs. familiar: *P* = 0.018, initial vs. novel: *P* = 0.004, Fig. 4*C*,*D*). Additionally, presentation of novel or familiar visual stimuli 24 h after HFS significantly decreased delta power in layers 4 and 5/6 (*n* = 11, RANOVA _(df, 2, 10)_ with Bonferroni post hoc. Layer 4: *F* = 10.24, *P* < 0.001; initial vs. familiar: *P* = 0.031, initial vs. novel: *P* < 0.001. Layer 5/6: *F* = 7.681, *P* = 0.003; initial vs. familiar: *P* = 0.04, initial vs. novel: *P* = 0.003, Fig. 4*D*).

### Visually Evoked Reset of Ongoing Gamma Oscillations Predicts Visual Response Potentiation

**Figure 5:**
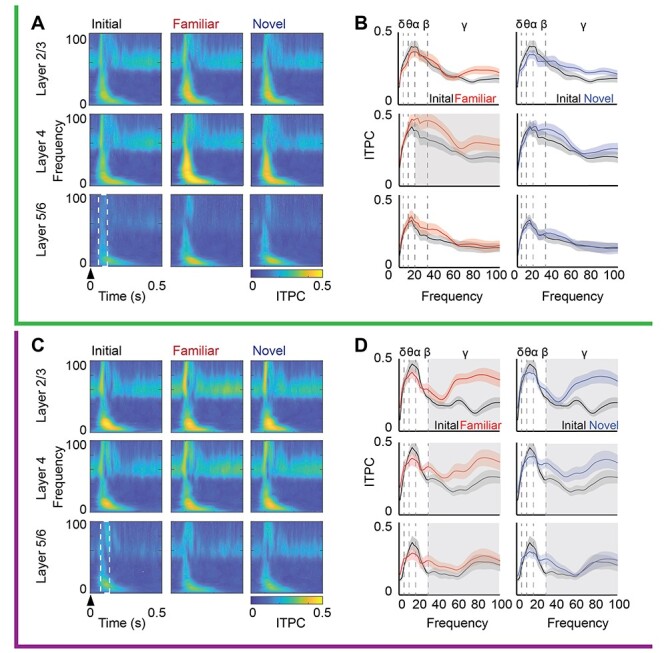
ITPC co-varies with LFS- and HFS-induced visual response potentiation. Top, green, after LFS: (*A*) Average ITPC 24 h after LFS in response to familiar and novel visual stimuli compared with initial response (ITPC; heat map) from 0 to 100 Hz (in 3 Hz bins; *y*-axis) over time (*x*-axis). Trial averaged from complex Morlet wavelet convolution. Arrowhead indicates stimulus onset; white box indicates time window for assessment of change in ITPC (100–200 ms after stimulus onset). (*B*) Average ITPC power binned by frequency band (δ: 1–4, θ: 4–8, α: 8–13, β: 13–30, γ: 30–100 Hz) in response to initial (black), familiar (red), and novel (blue) visual stimuli. Twenty-four hours after LFS, the familiar visual stimulus induced a significant increase phase reset of β and γ oscillations in layer 4 relative to initial response (black, RANOVA _(df, 2, 15)_, β: *F* = 5.20, *P* = 0.011, γ: *F* = 8.82, *P* < 0.001). Gray highlight = Bonferroni post hoc *P* < 0.05; *n* = 16 subjects. Bottom, purple, after HFS: (*C*) Average ITPC 24 h after HFS in response to familiar and novel visual stimuli, compared with initial response (ITPC; heat map) from 0 to 100 Hz (in 3 Hz bins; *y*-axis) over time (*x*-axis). Trial averaged from complex Morlet wavelet convolution. Arrowhead indicates stimulus onset; white box indicates time window for assessment of change in ITPC (100–200 ms after stimulus reversal). (*D*) Average ITPC power binned by frequency band (δ: 1–4, θ: 4–8, α: 8–13, β: 13–30, γ: 30–100 Hz) in response to initial (black), familiar (red), and novel (blue) visual stimuli. Twenty-four hours after HFS, familiar (red) and novel (blue) visual stimuli induced a significant increase in phase reset of γ oscillations in all cortical layers relative to initial (black, RANOVA _(df, 2, 10)_, layer 2/3: *F* = 19.42, *P* < 0.001; layer 4: *F* = 13.04, *P* < 0.001; layer 5: *F* = 5.29, *P* = 0.025). Gray highlight = Bonferroni post hoc *P* < 0.05; *n* = 11 subjects.

The phase of ongoing LFP oscillations, and the ability to reset phase with visual stimulation, can modify the amplitude of visually evoked responses. To ask if visual response potentiation is coincident with visually induced phase reset of the LFP, we calculated the average ITPC during maximum visually evoked activity (100–200 ms after stimulus onset). Twenty-four hours after LFS, the familiar visual stimulus significantly increased beta and gamma ITPC specifically in layer 4 (*n* = 16 subjects. RANOVA _(df, 2, 15)_, Bonferroni post hoc, β: *F* = 5.20, *P* = 0.011; initial vs. familiar: *P* = 0.045, γ: *F* = 8.82, *P* < 0.001; initial vs. familiar: *P* = 0.005, Fig. 5*A*,*B*). In contrast, 24 h after HFS, visual stimuli with both familiar and novel orientations significantly increased gamma ITPC in all cortical layers (*n* = 11 subjects. RANOVA _(df, 2, 10)_, Bonferroni post hoc, layer 2/3: *F* = 19.42, *P* < 0.001; initial vs. familiar: *P* = 0.001; initial vs. novel: *P* = 0.003. Layer 4: *F* = 13.04, *P* < 0.001; initial vs. familiar: *P* = 0.010; initial vs. novel: *P* = 0.006. Layer 5: *F* = 5.29, *P* = 0.025; initial vs. familiar: *P* = 0.022; initial vs. novel: *P* = 0.021, Fig. 5*C*,*D*). Notably, the expression of increased gamma ITCP mirrored the locus and specificity of VEP potentiation following LFS and HFS.

**Figure 6:**
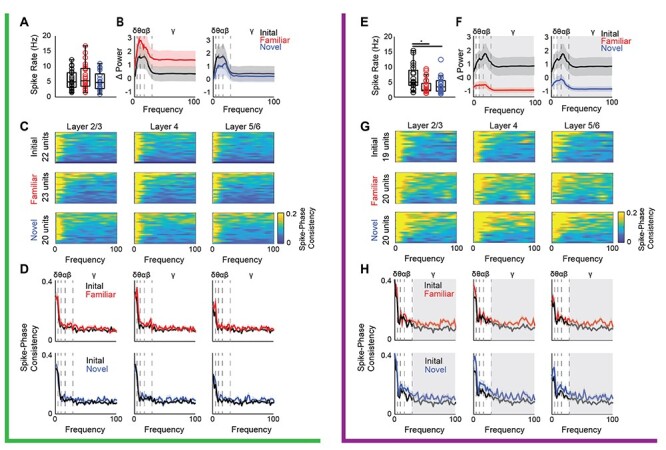
HFS decreases FS IN firing rates and power and increases FS IN spike–LFP gamma phase consistency. Top, green, after LFS: (*A*) No change in average spike rates of FS IN during presentation of familiar or novel visual stimuli 24 h after LFS. (*B*) No change in FS IN oscillatory power during presentation of familiar (red, top) or novel (blue, bottom) visual stimuli relative to initial (black). (*C*) Heat map of spike–phase consistency of FS INs during initial LFS, and familiar and novel visual stimuli 24 h after LFS by cortical layer from 0 to 100 Hz during the 100–200 ms following stimulus onset. (*D*) Average FS IN-LFP spike–phase consistency by cortical layer in response to initial (black), familiar (red), and novel (blue) stimuli. No significant difference in spike–phase consistency 24 h after LFS. Bottom, purple, after HFS: (*E*) 24 h after HFS, visual stimuli with familiar (red) and novel (blue) orientations significantly decreased average FS IN spike rate (ANOVA _(df, 2, 56)_, *F* = 4.50, *P* = 0.015). ^*^Tukey post hoc *P* < 0.05; *n* = 11 subjects. (*F*) Average change in FS IN oscillatory power by frequency during initial HFS (black) and familiar (red) and novel (blue) visual stimuli 24 h after HFS. A significant decrease in the oscillatory power of FS IN across multiple frequencies (7–100 Hz) during familiar and novel stimuli compared with initial (black; ANOVA_(df, 2, 56)_, α: *F* = 6.862, *P* = 0.002, β: *F* = 8.898, *P* = 0.0004, γ: *F* = 5.998, *P* = 0.004). Gray highlight = Bonferroni post hoc *P* < 0.05, *n* = 11 subjects. (*G*) Heat map of spike–phase consistency of FS IN during initial HFS, and familiar and novel visual stimuli 24 h after HFS by cortical layer from 0 to 100 Hz during the 100–200 ms following stimulus reversal. (*H*) Average FS IN spike–LFP phase consistency by cortical layer induced by initial (black), familiar (red), and novel (blue) stimuli. Twenty-four hours after HFS, familiar and novel visual stimuli induced a significant increase in FS IN spike–LFP γ phase consistency in all cortical layers (ANOVA_(df, 2, 56)_, layer 2/3: *F* = 6.795, *P* = 0.002; layer 4: *F* = 5.655, *P* = 0.006; layer 5: *F* = 6.072, *P* = 0.004). Gray highlight = Bonferroni post hoc *P* < 0.05; *n* = 11 subjects.

To ask if changes in FS IN activity reflect plasticity of visual responses, we examined the spontaneous spike rate and oscillatory power and evoked spike rate and oscillatory power in response to visual stimuli with familiar (60°) and novel (150°) orientations 24 h after LFS or HFS. Spontaneous FS IN firing rate and oscillatory power were suppressed 24 h after HFS (Supplementary Fig. S4F), but unchanged following LFS (Supplementary Fig. S4C). Similarly, there were no significant differences in visually evoked FS IN firing rate or power following LFS (*n* = 16 subjects, 22 (initial), 23 (familiar), 20 (novel) units; Fig. 6*A*,*B*). However, subsequent visual stimulation in subjects that received HFS significantly suppressed FS IN firing rates and significantly decreased the power of FS output at frequencies above theta (7–100 Hz; *n* = 11 subjects, 19 (initial), 20 (familiar), 20 (novel) units; firing rates: one-way ANOVA _(df, 2, 57)_, Tukey post hoc, *F* = 4.50, *P* = 0.015; initial vs. familiar: *P* = 0.022; initial vs. novel: *P* = 0.046, Fig. 6*E*; power: one-way ANOVA _(df, 2, 57)_, Bonferroni post hoc, α: *F* = 6.862, *P* = 0.002, initial vs. familiar: *P* = 0.004, initial vs. novel: *P* = 0.011, β: *F* = 8.898, *P* < 0.001, initial vs. familiar: *P* = 0.003, initial vs. novel: *P* = 0.001, γ: *F* = 5.998, *P* = 0.004, initial vs. familiar: *P* = 0.009, initial vs. novel: *P* = 0.015; Fig. 6*F*). We observed no change in the spontaneous spike rates of RS neurons 24 h after LFS and a significant increase in visually evoked RS spike rate in layer 4 in response to the familiar orientation, as previously reported (Aton et al. 2014, *n* = 16 subjects, 33 (initial), 28 (familiar), 25 (novel) units: one-way ANOVA_(df, 2, 83)_ Tukey post hoc, *F* = 3.16, *P* = 0.047; initial vs. familiar: *P* = 0.037, data not shown). Twenty-four hours after HFS, RS units in layer 5 demonstrated a significant decrease in spontaneous firing rates (*n* = 11 subjects, 20 (0 h), 15 (24 h) units. Student’s *t*-test, *t* = 2.63, *P* = 0.0065) and evoked firing rates to familiar and novel orientations (*n* = 11 subjects, 20 (initial), 17 (familiar), 20 (novel) units: one-way ANOVA_(df, 2, 54)_, Tukey post hoc, *F* = 6.24, *P* = 0.0036; initial vs. familiar: *P* = 0.014, initial vs. novel: *P*= 0.007, data not shown).

To ask how visually evoked changes in FS IN firing are related to the phase of ongoing LFP oscillatory activity, we calculated spike-phase consistency between each FS IN and the LFP of each cortical layer. Twenty-four hours after LFS, there was no significant change in FS IN spike–LFP–phase consistency in any cortical layer in response to novel or familiar visual stimuli (*n* = 16 subjects, 22 (initial), 23 (familiar), 20 (novel) units; Fig. 6*C*,*D*). In contrast, 24 h after HFS, visual stimuli with familiar and novel orientations significantly increased FS IN spike–LFP phase coupling with gamma oscillations across all cortical layers (*n* = 11 subjects, 19 (initial), 20 (familiar), 20 (novel) units. One-way ANOVA_(df, 2, 56)_, Bonferroni post hoc. Layer 2/3: *F* = 6.795, *P* = 0.002, initial vs. familiar: *P* = 0.009, initial vs. novel: *P* = 0.005. Layer 4: *F* = 5.655, *P* = 0.033, initial vs. familiar: *P* = 0.008, initial vs. novel: *P* = 0.005. Layer 5: *F* = 6.072, *P* = 0.004, initial vs. familiar: *P* = 0.026, initial vs. novel: *P* = 0.006; Fig. 6*G*,*H*). Thus, visual stimulation subsequent to HFS decreases FS IN firing rates, and increases FS IN phase coupling with gamma, throughout the primary visual cortex.

### HFS Enhances Visual Acuity

**Figure 7:**
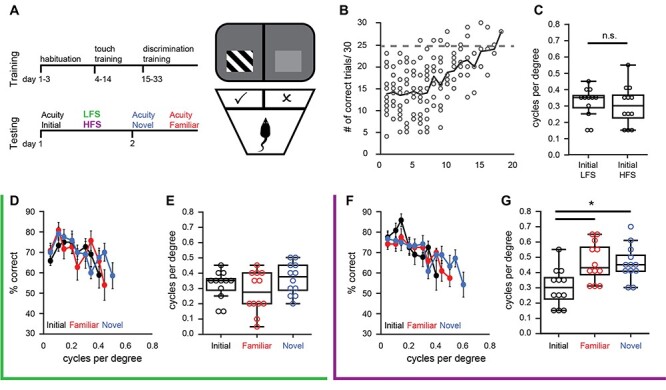
HFS enhances visual acuity. (*A*) Left: Timeline, subjects were trained in a two-alternative forced choice visual detection task until they reached criterion of 25/30 correct choices at 0.05 cpd. Baseline visual acuity was assessed using a novel stimulus orientation and followed by LFS or HFS. Spatial acuity was tested again 24 h after LFS or HFS. Right: Cartoon depicting modified Bussey chamber with plexiglass divider to define the choice point for the determination of stimulus spatial frequency. (*B*) All subjects reached task criterion by 18 days of training. (*C*) No significant difference in initial visual acuity prior to the delivery of LFS or HFS (Student’s *t*-test, *P* = 0.63). (*D*) Average frequency of seeing curves for subjects that received LFS (green box). (*E*) No significant difference in spatial acuity probed with initial (black, before LFS), novel (blue, after LFS), and familiar (red, after LFS) visual stimulus orientations (*n* = 12). (*F*) Average frequency of seeing curves for subjects that received HFS (purple box). (*G*) A significant increase in spatial acuity probed with novel (blue, after HFS) and familiar (red, after HFS) visual stimulus orientations following HFS (black, before HFS; RANOVA _(df, 2, 11)_, *F* = 6.817, *P* = 0.005). ^*^Bonferroni post hoc *P* < 0.05; *n* = 12.

Following HFS, an increase in VEP magnitudes and ITPC is observed in response to visual stimuli with novel orientations and spatial frequencies, suggesting that HFS may generally enhance visual acuity. To test this prediction, we examined the impact of LFS and HFS on spatial acuity assessed by performance in a two-alternative forced-choice spatial frequency detection task utilizing a Bussey-Saksida touch screen hamber with a plexiglass insert to define the choice point for calculation of the visual stimulus spatial frequency. Naïve mice (*n* = 12) were trained to associate a liquid reward with a simple visual stimulus (high contrast (100%), low spatial frequency (0.05 cpd) 45° sinusoidal grating; Fig. 7*A*). Subjects performed 30 trials per day, requiring 12.3 ± 1.05 days to reach the criterion of 25/30 correct trials (83%; Fig. 7*B*). To assess spatial acuity, the positive stimulus was rotated to a novel orientation (45° ± 15°), and subjects completed blocks of 10 trials with spatial frequencies from 0.05 to 0.7 cpd in increments of 0.05 cpd. Spatial acuity was defined as highest spatial frequency with performance of ≥70% correct choices.

Following determination of baseline spatial acuity, subjects passively viewed 200 s of either LFS or HFS at a novel orientation (45° ± 30°). There was no significant difference in baseline spatial acuity between subjects assigned to LFS and HFS groups (average ± SEM cpd, LFS: 0.33 ± 0.02; HFS: 0.30 ± 0.03 cpd, Student’s *t*-test, *P* = 0.63, Fig. 6*C*). Twenty-four hours following visual stimulation, visual performance was assessed at the familiar (45° ± 30°) or another novel orientation (45° ± 60°). Following LFS, we observed no change in spatial acuity assessed with the familiar (0.30 ± 0.03 cpd) or novel visual stimulus (0.37 ± 0.03 cpd; *n* = 12, RANOVA _(df, 2, 11)_, *F* = 4.02, *P* = 0.032, initial vs. familiar: *P* = 0.303, initial vs. novel: *P* = 0.303; Fig. 7*D*,*E*). In contrast, 24 h after HFS, spatial acuity was significantly enhanced in response to familiar (0.46 ± 0.03 cpd) and novel visual stimulus orientations (0.46 ± 0.03; *n* = 12, RANOVA _(df, 2, 11)_, Bonferroni post hoc, *F* = 6.87, *P* = 0.005; initial vs. familiar: *P* = 0.011; initial vs. novel: *P* = 0.011; Fig. 7*F*,*G*). Together, this suggests that HFS induces a sustained and highly generalizable enhancement of visual function.

## Discussion

Distinct cellular, circuit and perceptual consequences are induced in the mouse visual system by high (HFS) and low (LFS) frequency repetitive visual stimulation. A single, short bout of LFS was sufficient to engage selective response potentiation of layer 4 VEPs, which mirrored stimulus-selective response potentiation induced by daily LFS in specificity, locus of expression, and the absence of change in FS IN output (Frenkel et al. 2006; Aton et al. 2014). In contrast, a single bout of HFS induced a long-lasting suppression of the output of FS INs and primed high-frequency (gamma) LFP oscillations for subsequent visually evoked phase reset. Accordingly, we show that subsequent to HFS, VEP amplitudes are potentiated in all layers of V1 and in response to both familiar and novel visual stimuli. Together this demonstrates that the temporal characteristics of repetitive visual stimulation are a decisive factor in the locus of expression and the specificity of visual response plasticity.

It is increasingly appreciated that activity in V1 neurons reflects more than visual input. Indeed, neuronal activity patterns are altered by locomotion (Niell and Stryker 2010), generalized motor movements, arousal state (Stringer et al. 2019), and input from other sensory systems (Iurilli et al. 2012). Similarly, ongoing cortical rhythms in V1, including beta (12–30 Hz) and gamma (30–100 Hz), are regulated by visual stimulus parameters including contrast intensity (Saleem et al. 2017) and visual stimulus size (Veit et al. 2017). High-frequency and low-frequency sensory stimulation can acutely impact discrimination thresholds and induce acute desynchronization of alpha oscillations that last for up to 1 h (Clapp et al. 2006). Importantly, changes in cortical oscillatory power induced by bouts of visual stimulation across days encode complex temporal sequences (Han et al. 2008; Gavornik and Bear 2014) and reward timing (Zold and Hussain Shuler 2015). Similarly, visual stimulus familiarity is encoded by an increase in the visually evoked power of theta (4–8 Hz), alpha (8–12 Hz), and beta (12–30 Hz) oscillations in V1 layer 4 of V1 (Kissinger et al. 2018; Kissinger et al. 2020). We observed an increase in mid-range (alpha and beta) oscillatory activity in V1 in response to a familiar visual stimulus just 24 h after a single bout of LFS. However, 24 h after a single bout of HFS, low-range (theta) power was decreased in all cortical layers in response to both familiar and novel visual stimuli. The latter is consistent with our observation that HFS suppresses the firing rate of FS INs, as FS INs have been shown to drive the production of theta oscillations within the cortex and hippocampus (Buzsáki 2002; Stark et al. 2013). Twenty-four hours after HFS, we also observe a decrease in visually evoked and spontaneous low-frequency power (1–8 Hz) in all cortical layers in response to novel and familiar stimuli, as seen during directed attention (Schroeder and Lakatos 2009). Thus, LFS and HFS have distinct long-term consequences on LFP oscillatory activity in V1.

Changes in the power of cortical oscillations in the absence of synchronization of oscillatory phase could reduce response magnitude by increasing variability. In contrast, phase synchronization with incoming stimulation could increase response magnitude and decrease response variability. Indeed, co-incident visually induced phase reset of cortical gamma oscillations predicted both the location and specificity of visual response potentiation in response to LFS and HFS. Both familiar and novel visual stimuli, subsequent to HFS, reset the phase of ongoing gamma oscillations throughout V1, while visually induced gamma phase reset following LFS is selective for layer 4 and the familiar visual stimulus. The ability to prime high-frequency oscillatory activity likely reflects the origins of generation and modulation of gamma oscillations. Cortical gamma oscillations are generated by feedforward thalamocortical connections, while phase synchrony is modulated/entrained by the output of cortical parvalbumin expressing FS INs (Cardin et al. 2009; Chen et al. 2017; Saleem et al. 2017). HFS-induced suppression of FS INs would reduce the impact of cortical activity on gamma synchrony and enhance sensitivity to gamma phase reset by subsequent visually evoked feed-forward activity. Accordingly, the nonselective response potentiation we observe following HFS is reminiscent of the enhancement of visually evoked responses to both familiar and novel visual stimuli following optogenetic suppression of FS IN output (Kaplan et al. 2016).

LFS and HFS are also likely to recruit activity in different subsets of neurons in mouse V1, tuned to lower and higher temporal frequencies, respectively (Gao et al. 2010). Nevertheless, the temporal frequency of the initial visual stimulation was reflected in changes in LFP oscillatory power, phase reset, and FS IN output. The temporal frequency of the HFS used here (20 Hz) is close to flicker fusion in the murine visual system and may therefore drive the largest number of pyramidal neurons to spike at high frequency (Tanimoto et al. 2015; Durand et al. 2016). Interestingly, the suppression of FS IN firing rates and changes in synchrony of FS IN spiking with oscillations are observed 24 h after HFS but not during HFS. One likely possibility is that sleep is required to consolidate changes in cortical activity induced by HFS, as has been shown for the stimulus-selective response potentiation induced by daily LFS (Aton et al. 2014).

Our results suggest that the expression site and generalizability of response potentiation are determined by the temporal frequency of the inducing stimulus. The LFS and HFS stimulation protocols used here were matched for total duration, as such HFS subjects received more contrast reversals than those that received LFS. Although we cannot rule out the possibility that the difference in the number of stimulus presentations, and not the temporal frequency of the visual stimulus, underlies the response potentiation following HFS, previous studies have shown that daily repetition of LFS induces a robust response potentiation that is stimulus-specific and expressed in layer 4 (Frenkel et al. 2006; Cooke and Bear 2010). This suggests that increasing the number of LFS stimulus presentations would not impact the locus or specificity of response potentiation. Yet, the response potentiation induced by our truncated LFS paradigm mirrored the response to daily LFS in specificity and locus of expression but was more modest in amplitude. It is worth noting that although our initial VEP amplitudes were low compared with previous work utilizing single tungsten electrodes in layer 4 (Frenkel et al. 2006), they are consistent with our previous work utilizing a laminar array of small diameter platinum iridium wires (Murase et al. 2017). Additionally, we cannot rule out the possibility that the synaptic changes underlying improved spatial acuity following HFS reflect plasticity beyond V1, such as the laterointermediate area, where neurons are tuned to higher temporal and spatial frequencies than V1 (Marshel et al. 2011). However, perceptual habituation induced by daily LFS transfers from training to testing environments and shares the stimulus selectivity and dependence on NMDARs located in V1 NMDARs (Cooke et al. 2015). Our visual acuity measures are the first to utilize a modified Bussey Chamber (Horner et al. 2013) to which we added a plexiglass swing-through door to identify a choice point for visual stimulus spatial frequency calculation. Our acuity measurements are lower than those previously reported using a water-based task (Prusky et al. 2000) and may reflect the rapid acquisition of the touchscreen visual detection task with a minimal number of trials, or that spatial frequency detection is occurring prior to the arrival at the plexiglass threshold. Nonetheless, our acuity measures are highly consistent within subjects, and only subjects that received HFS stimulation demonstrated improved visual detection.

Our findings provide mechanistic insight into the distinct cellular, circuit, and perceptual response to high (HFS) and low (LFS) frequency repetitive visual stimulation, which may lend insight to other forms of sensory system plasticity and to other species. Indeed, cortical oscillations, as well as their functions, are highly conserved across species within multiple spatiotemporal scales, remaining consistent even with increasing brain size (Buzsáki et al. 2013). In humans, a high-frequency (20 Hz) luminance flicker induces a long-lasting improvement in luminance change detection, while a low-frequency (1 Hz) luminance flicker briefly reduces performance (Beste et al. 2011). Similarly, high frequency (20 Hz), but not low frequency (1 Hz), presentation of an oriented bar or a flickering sinusoidal grating improves orientation discrimination (Marzoll et al. 2018). However, the optimal frequency of stimulation to improve visual function may not be consistent across species. Indeed, visual stimulation with a 9-Hz tetanus in humans improves detection thresholds but fails to potentiate VEP amplitudes in rodents (Teyler et al. 2005; Clapp et al. 2012; Eckert et al. 2013; but see Abuleil et al. 2019). Performance on sensory and memory tasks is also manipulated by transcranial brain stimulation techniques. Low-frequency repetitive transcranial magnetic stimulation (rTMS) (1–3 Hz pulses) stimulation over the visual cortex in cats induced transient depression of the amplitude of visual response amplitudes, while HFS (10 Hz) transiently potentiated visual responses (Aydin-Abidin et al. 2006). Together this supports the continued evaluation of HFS visual stimulation for noninvasive vision therapy to promote long-lasting and generalizable enhancement of visual function.

## Supplementary Material

LantzQuinlan2021_CCComms_Supplement_tgab016
