# Supplementary material for: High-Frequency Visual Stimulation Primes Gamma Oscillations for Visually Evoked Phase Reset and Enhances Spatial Acuity
Source: Cereb Cortex Commun. 2021 Mar 3;2(2):tgab016. doi: 10.1093/texcom/tgab016 (PMC8110461; doi:10.1093/texcom/tgab016)
Supplement: LantzQuinlan2021_CCComms_Supplement_tgab016 [file lantzquinlan2021_cccomms_supplement_tgab016.pdf]

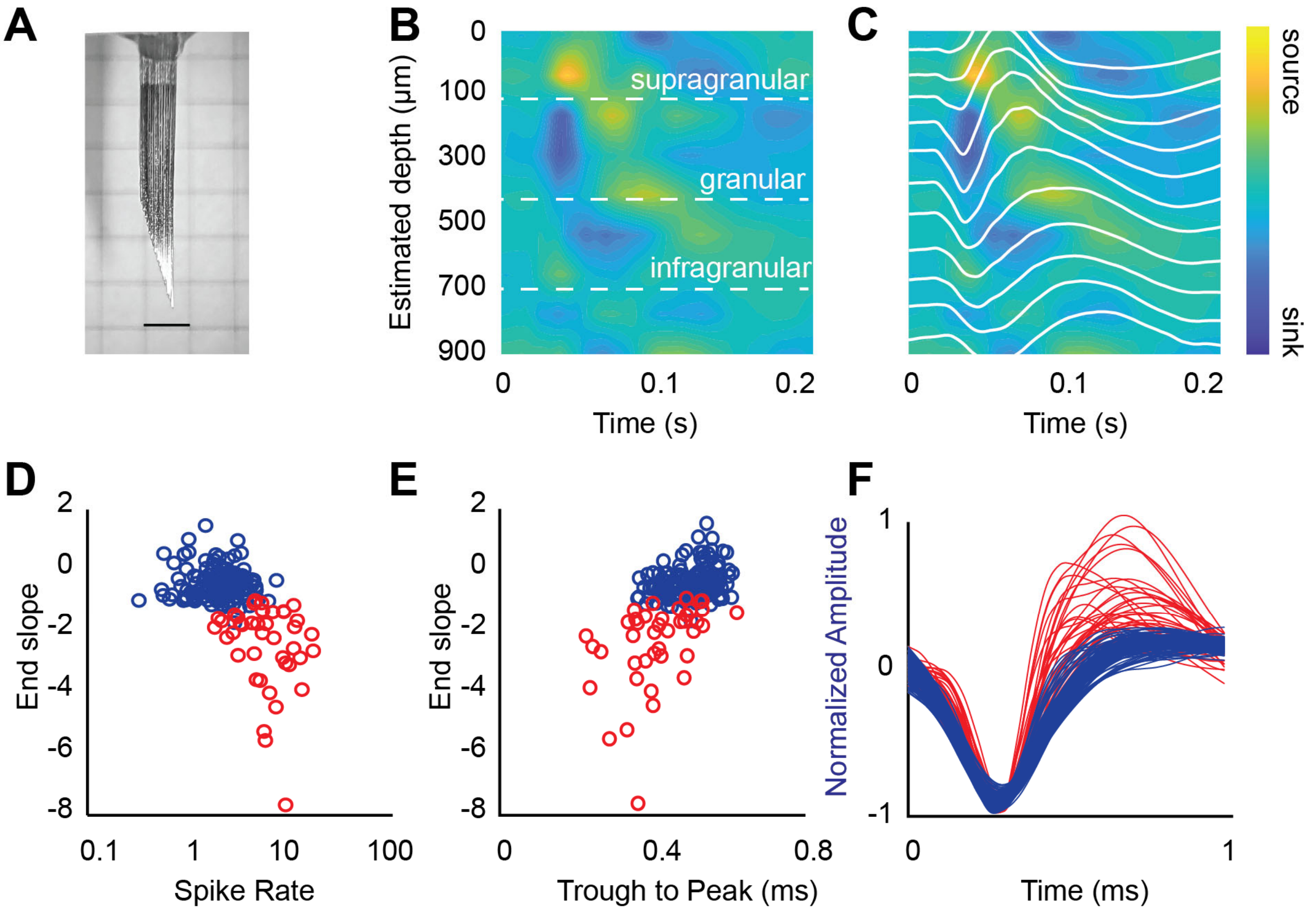

Figure S1. Assignment of electrode by layer and assessment of SU waveform. A) Representative photograph of electrode array, scale bar = 0.5 mm. B) Average current source density (CSD) depicting early layer 4 thalamocortical input current sink at 300 µm in blue, with estimated cortical depth. C) Overlay of average VEP waveforms used to calculate the CSD aligned with estimated cortical layer. D) Sorted RS (blue) and FS IN (red) plotted by spike rate and end slope of each average waveform. E) Scatter plot of RS (blue) and FS (red) single units based on end slop and trough to peak duration. F) Single unit waveforms of RS (blue) and FS IN (red) units.

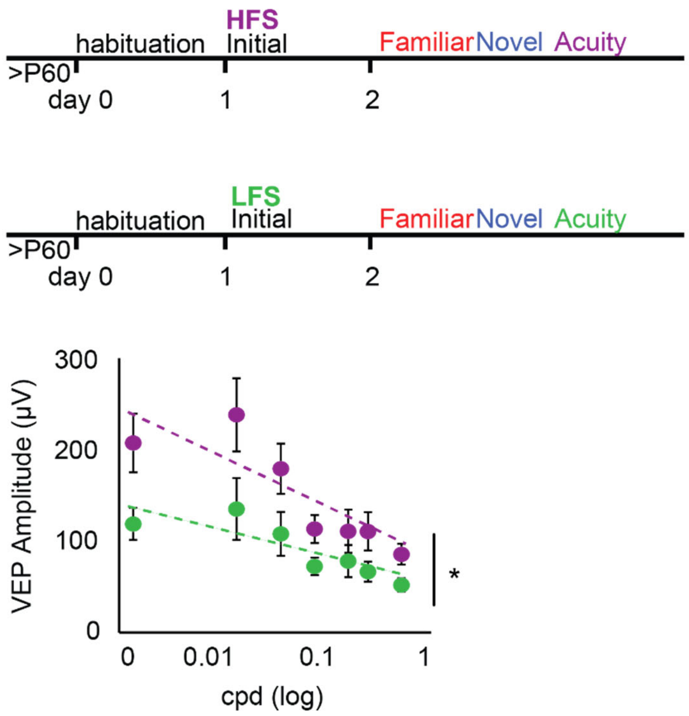

Figure S2. HFS enhances VEP amplitudes evoked by novel spatial frequencies. 24 hours after HFS, a significant increase in layer 4 VEP amplitudes is observed in response to visual stimuli with a novel orientation, across a range of spatial frequencies (purple) compared to LFS. (Between groups $RANOVA_{(df,6,1)}$, $F = 5.88$, $p = 0.035$). * = $p < 0.05$; n = 9 (HFS), 6 (LFS) subjects.

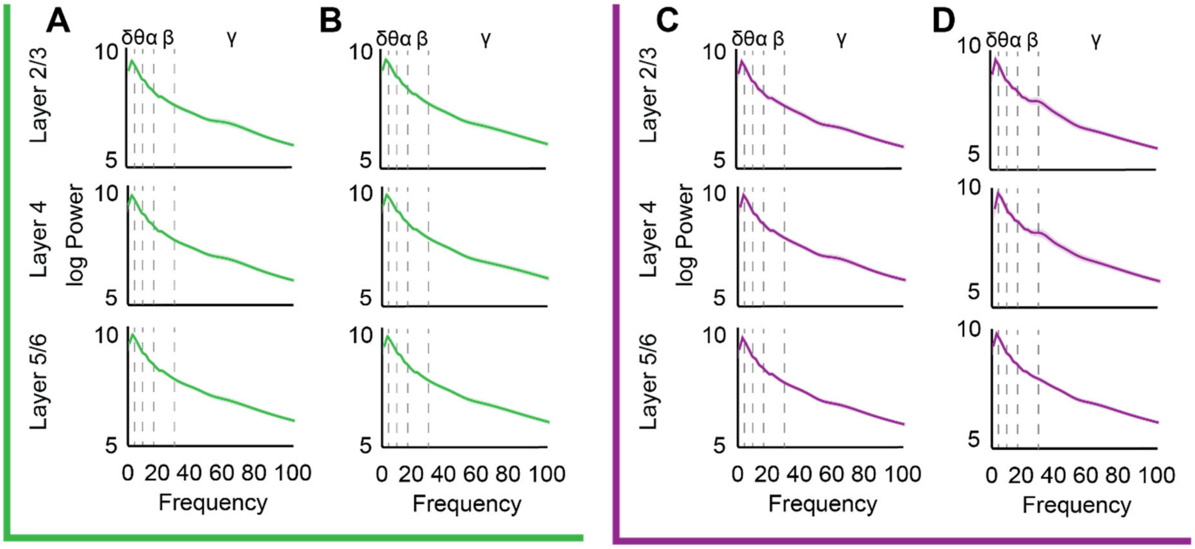

Figure S3. Non-normalized power distribution pre-visual stimulus, and during LFS or HFS. Left, green, LFS subjects, n = 16: A) Spontaneous cortical oscillatory power pre-LFS by cortical layer (average +/- SEM). B) Cortical oscillatory power during LFS by cortical layer (average +/- SEM). Right, purple, HFS subjects, n = 11: C) Spontaneous cortical oscillatory power pre-HFS by cortical layer (average +/- SEM). D) Cortical oscillatory power during HFS by cortical layer (average +/- SEM).

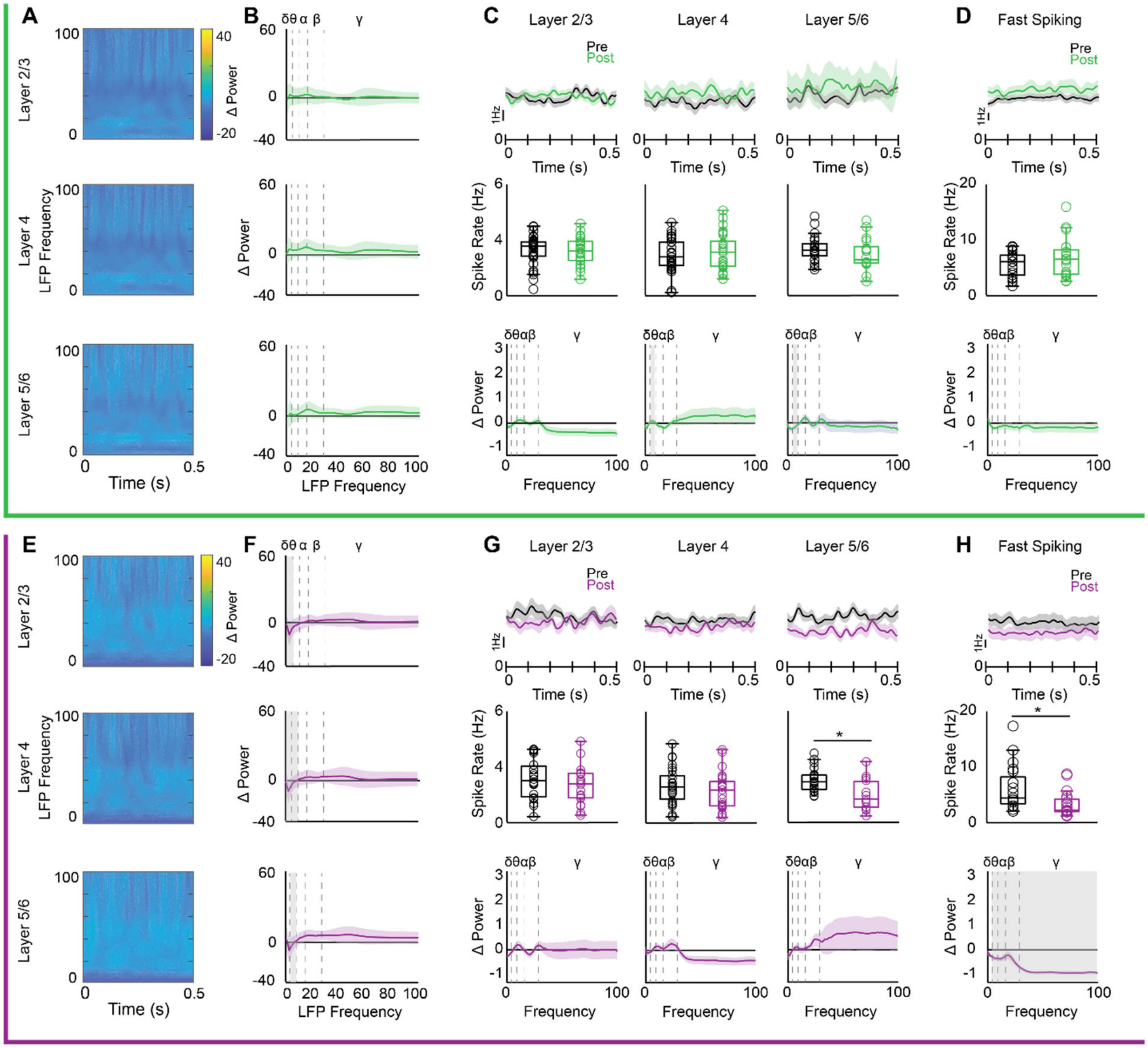

Figure S4. Distinct sustained effects of LFS and HFS on spontaneous oscillatory power and FS IN spiking. Top, green, 24 hours after LFS: A) Heat map, average change in spontaneous oscillatory power 24 hours after LFS (% change from pre-stimulation spontaneous activity) from 0-100 Hz (3 Hz bins; y axis) over time (x axis) by cortical layer. B) Average % change in spontaneous oscillatory power 24 hours after LFS, binned by frequency band (δ: 1-4, θ: 4-8, α: 8-13, β: 13-30, γ: 30-100 Hz). No change in spontaneous oscillatory power 24 hours after LFS in any cortical layer (n = 16). C) Top, average +/- SEM time-histogram of FS INs, pre (black) and 24 hours post LFS (green). Middle, average FS IN spike rates pre (black) and 24 hours post LFS (green). Bottom, no change in oscillatory power of FS IN spontaneous activity 24 hours post LFS, binned by frequency band. A significant decrease in θ, β, and γ oscillatory power 24 hours after LFS. Bottom, purple, 24 hours after HFS: D) Heat map, average change in spontaneous oscillatory power 24 hours after HFS (% change from pre-HFS spontaneous activity) from 0 to 100 Hz (3 Hz bins; y axis) over time (x axis) by cortical layer. E) Average % change in spontaneous oscillatory power 24 hours after HFS, binned by frequency band. 24 hours after HFS, δ power is significantly decreased in all layers (one sided t-test, grey highlight = $p < 0.05$; n = 11). F) Top, average +/- SEM time-histogram of FS INs pre LFS (blank) and 24 hours post HFS (purple). Middle, Significant decrease in average FS IN firing rates 24 hours after HFS (unpaired t-test, * = $p < 0.05$; n = 19 (pre), 18 (post) units, 11 subjects). Bottom, average change in spontaneous oscillatory power of FS INs 24 hours post LFS, binned by frequency band. Significant decrease in all frequency bands above δ 24 hours after HFS (One-sided t-test, grey highlight = $p < 0.05$; n = 18 units, 11 subjects).

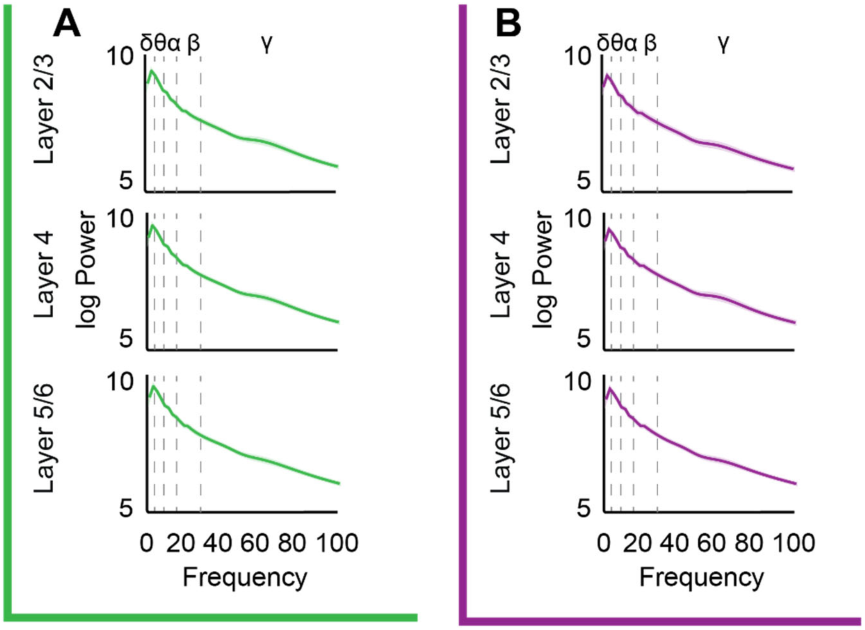

Figure S5. Non-normalized power distribution 24 hours after LFS or HFS. Left, green, LFS subjects, n = 16: A) Spontaneous oscillatory power during 24 hours after LFS by cortical layer (average +/- SEM). Right, purple, HFS subjects, n = 11: B) Spontaneous cortical oscillatory power 24 hours after HFS by cortical layer (average +/- SEM).
